# FITC and Ru(phen)_3_^2+ ^co-doped silica particles as visualized ratiometric pH indicator

**DOI:** 10.1186/1556-276X-6-561

**Published:** 2011-10-25

**Authors:** Jianquan Xu, Lei Sun, Jun Li, Jinglun Liang, Huimao Zhang, Wensheng Yang

**Affiliations:** 1 State Key Laboratory of Supramolecular Structure and Materials, College of Chemistry, Jilin University, Changchun 130012, People's Republic of China; 2 College of Public Health, Jilin University, Changchun, Jilin, 130021, People's Republic of China; 3 China-Japan Union Hospital, Jilin University, Changchun 130033, People's Republic of China

**Keywords:** pH indicator, visualized, silica particles, ratiometric, fluorescein, ruthenium complex

## Abstract

The performance of fluorescein isothiocyanate (FITC) and tris(1, 10-phenanathroline) ruthenium ion (Ru(phen)_3_^2+^) co-doped silica particles as pH indicator was evaluated. The emission intensity ratios of the pH sensitive dye (FITC) and the reference dye (Ru(phen)_3_^2+^) in the particles were dependent on pH of the environment. The changes in emission intensity ratios of the two dyes under different pH could be measured under single excitation wavelength and readily visualized by naked eye under a 365-nm UV lamp. In particular, such FITC and Ru(phen)_3_^2+ ^co-doped silica particles were identified to show high sensitivity to pH around the pKa of FITC (6.4), making them be potential useful as visualized pH indicator for detection of intracellular pH micro-circumstance.

## Background

In recent years, ratiometric fluorescent pH indicators had been developed for sensitive detection of pH of an analyte [1-6]. To fabricate a ratiometric pH indicator, usually two dyes, one pH sensitive and one reference dyes, were incorporated into a silica or polymer matrix. In this approach, a core/shell architecture in which the reference dye was mainly located in the core and the pH-sensitive dye located primarily in the shell was preferred [2,7]. The ratios in emission intensity of the two dyes were correlated to pH of the analyte. Compared to pH indicator containing only the pH-sensitive dye [8-13], such ratiometric pH indicator was more reliable since the ratios in emission intensity were less sensitive to the fluctuations in excitation light source intensity and variations in other experimental conditions except pH [3,4,14-16]. However, most of the ratiometric pH indicators reported required the measurements of the emission intensity of the two dyes under two different excitation wavelengths, which made the analysis process be complicated and difficult to be visualized by naked eye [2,4,6,7,17].

In our previous work, we developed a kind of multicolor silica particles co-doped by fluorescent (fluorescein isothiocyanate - FITC) and phosphorescent (Ru(phen)_3_^2+^) dyes. The green FITC and red Ru(phen)_3_^2+ ^dyes could be synchronously excited by a single excitation wavelength since there was large overlapping region in their absorption spectra. Color of the dye-doped silica particles was tunable by simply the ratios of the two dyes, which was readily visualized under a 365-nm UV lamp by naked eye [18]. In this work, we explored the feasibility of such FITC and Ru(phen)_3_^2+ ^co-doped silica particles as visualized pH indicator, in which the green FITC was used as the pH sensitive dye and the red Ru(phen)_3_^2+ ^was employed as reference dye. It is expected that the particles may present different colors under different pH since the emission intensity of FITC was sensitive to pH. Experimental results revealed that the particles showed visualized color changes from red to yellowish-green distinguishable under a 365-nm UV lamp when pH of the buffer solutions increased from 2 to 8. Specially, such ratiometric pH indicator was very sensitive to pH around the pKa of FITC (6.4), making it potential useful for detection of intracellular pH micro-circumstance.

## Experimental section

### Materials

FITC, 3-aminopropyltriethoxysilane (APS), and dichloro tris (1,10-phenanathroline) ruthenium (II) hydrate (Ru(phen)_3_^2+^) were purchased from Aldrich Chemical Co. (Milwaukee, WI, USA). Tetraethoxysilane (TEOS, Tiantai Chemical Int., Tianjin, China) was distilled under reduced pressure before use. Analytical grade ethanol, ammonia hydroxide (25%), NaOH (98%), H_3_PO_4 _(85%), H_3_BO_3 _(99%), and CH_3_COOH (36%) were purchased from Beijing Chemical Int. (Beijing, China) and used without further purification. Dulbecco's Modified Eagle Medium (DMEM), fetal bovine serum (FBS), and phosphate-buffered saline (PBS) were purchased from Invitrogen Gibco Corp. (Carlsbad, CA, USA). The human hepatoma cell line SMMC-7721 was purchased from Cell Resource Center of Shanghai Institutes for Biological Sciences (Shanghai, China). Britton-Robinson buffer solutions (denoted as BR buffer solution hereafter) in the pH range of 2.0-10 were prepared from a solution containing H_3_BO_3_, H_3_PO_4_, and CH_3_COOH with the same concentration of 0.04 mol L^-1^, and the desired pH value were acquired by adding different volume of 0.2 mol L^-1 ^of NaOH. High-purity water with a resistivity of 18.2 MΩ cm (Pall Purelab Plus) was used in all experiments.

### Synthesis of Ru(phen)_3_^2+^-doped silica particles

Ru(phen)_3_^2+^-doped silica particles were prepared by a modified Stöber method. In a typical reaction, 3 mL TEOS was added to ethanol solution (60 mL) containing ammonia (2.4 mL), Ru(phen)_3_^2+ ^(0.6 mg, dissolved in 1 mL ethanol), and water (1.2 mL). The reaction mixture was kept at 40°C for 6 h, then another 0.8 ml TEOS was added for the growth of an additional silica layer, and then the reaction was continued for another 6 h. The reaction solution was centrifuged at 10,000 rpm for 15 min to collect the silica particles. The particles were further washed with ethanol for three times to remove the unreacted chemicals and then dispersed in 60 mL ethanol.

### Synthesis of FITC and Ru(phen)_3_^2+ ^co-doped silica particles

Ammonia (2.4 mL) and water (1.2 mL) were added into the ethanol dispersion of the Ru(phen)_3_^2+^-doped silica particles (60 mL) and then 80 μL APS was added into the mixture. After being kept at 40°C under magnetic stirring for 8 h, the reaction solution was centrifuged at 10,000 rpm for 15 min to collect the aminated silica particles. After being washed three times with ethanol to remove the unreacted chemicals, the particles were dispersed to 60 mL ethanol and then 1 mg FITC dissolved in 1 mL ethanol was added. The mixture was allowed to stand at 40°C under magnetic stirring for 12 h. After the reaction, the particles were centrifuged at 10,000 rpm for 15 min to remove the unreacted dyes. The particles were washed by water until no fluorescence was detectable in the supernatant.

### Cell handing process

SMMC-7721 cells were cultured in DMEM containing 10% FBS (fetal bovine serum) with 100 U/ml penicillin and 100 μg/ml streptomycin and incubated at 37°C under a humidified atmosphere containing 5% CO_2_. The cells were seeded in culture plates at a density of 1 × 10^5 ^cell/mL. After 24-h culturing, the cells were treated with the as-prepared silica particles which dispersed in serum-free DMEM at a concentration of 100 μg/mL for 4 h. After treatment, the cells were isolated by trypsin and washed with PBS for three times, and then the cells after endocytosis of the silica particles were observed by a fluorescence microscopy.

### Characterizations

Transmission electron microscopic (TEM) observations were carried out on a JEOL-2010 electron microscope (JEOL, Tokyo, Japan) operating at 200 kV for determining the sizes of silica particles. The samples were prepared by depositing a drop of the dispersion of the particles onto carbon grids (200 mesh) and allowing evaporation of the solvent in air at room temperature. Emission spectra were measured on an Edinburgh FS900 steady-state fluorescence spectrometer (Edinburgh Instruments Ltd., Livingston, UK) with a 450-W xenon lamp as excitation source. Absorption spectra were collected with a Varian Cary-100 scan UV-vis spectrophotometer. Fluorescence images were taken under a 400 times OLYMPUS IX71 fluorescence microscope excited at 450 nm.

## Results and discussion

**Figure 1:**
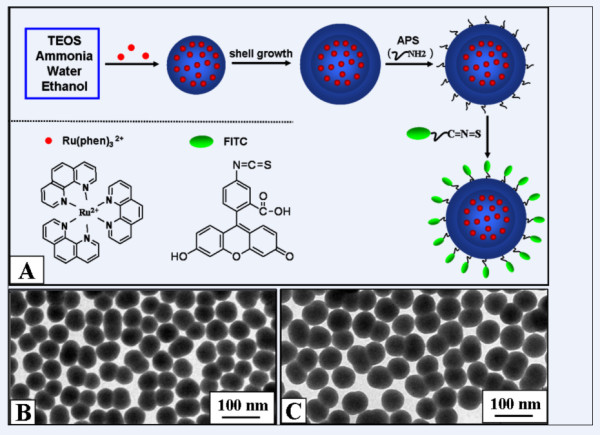
**Preparation of FITC and Ru(phen)_3_^2+ ^co-doped silica particles and TEM images of the Ru(phen)_3_^2+^-doped particles**. **(A) **Procedures for preparation of the FITC (green) and Ru(phen)_3_^2+ ^(red) co-doped silica particles. TEM images of the Ru(phen)_3_^2+^-doped particles **(B) **before and **(C) **after the shell growth and graft of FITC.

**Figure 2:**
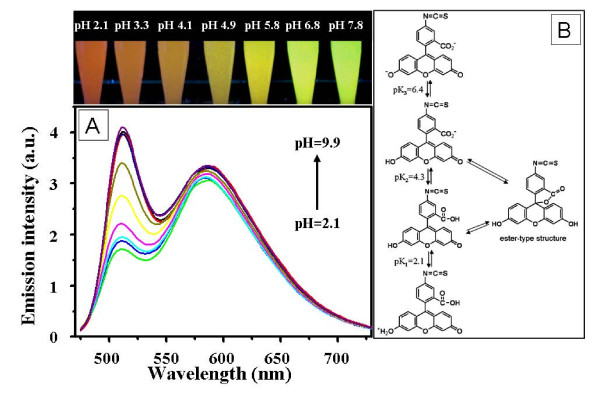
**Emission spectra of the co-doped silica particles and molecular structures of FITC under different pH**. **(A) **Emission spectra of the co-doped silica particles dispersed in BR buffers with pH of 2.1, 3.3, 4.1, 4.9, 5.8, 6.8, 7.8, 8.9, and 9.9. The excitation wavelength was at 450 nm. Insert gives the photos of the particles dispersed in BR buffers with different pH under a 365 nm UV lamp. **(B) **Molecular structures of FITC under different pH.

Figure 1A illustrates the procedures for preparation of the FITC and Ru(phen)_3_^2+ ^co-doped silica particles. First, the reference dye, Ru(phen)_3_^2+^, was incorporated into the silica particles by electrostatic adsorption via the modified Stöber method [19-21]. Average diameter of the silica particles was determined to be 52 nm as indicated by TEM observations (Figure 2B). After the centrifugation treatment, no emission of the dye was detectable in the supernatant, indicating complete incorporation of the reference dye added into the silica particles. After the growth of a silica shell, surface of the Ru(phen)_3_^2+ ^silica particles was functionalized by amino groups. FITC was grafted onto surface of the aminated particles by formation of covalent bond between the amino groups on the particle surface and isothiocyanate group of FITC. The ratio of FITC and Ru(phen)_3_^2+ ^in the particles could not be determined directly from the absorption spectrum of the co-doped silica particles since there was large overlap between their absorption features (see Figure S1 of Additional file 1). Pure silica particles (52 nm) without Ru(phen)_3_^2+ ^were adopted to evaluate the labeling efficiency of FITC. After graft of FITC onto the pure silica particles, the particles were centrifuged and washed with water until the supernatant was clear. Then the particles were dissolved in 0.5 M NaOH solutions to liberate the dye molecules [22]. The labeling efficiency of FITC was estimated to be about 47% from the absorption spectra (see Figure S2 of Additional file 1). So the actual molar ratio of Ru(phen)_3_^2+ ^and FITC in the silica particles was about 2:3. In such co-doped silica particles, the reference dye molecules were mainly located in the core part of the particles to prevent their direct contact with the solvent environment. On the contrary, the pH sensitive dye molecules were primarily located on the surface of the particle to maximize their contact with the analyte. The as-prepared FITC and Ru(phen)_3_^2+ ^co-doped silica particles were well dispersed in aqueous solution. Average diameter of the co-doped particles was determined to be 60 nm as observed by TEM (Figure 1C). It was deduced that the shell thickness was about 4 nm since the fictionalization of APS and FITC had little effect on the particle size.

Figure 2A shows the emission spectra of the co-doped silica particles dispersed in BR buffer solutions with different pH. The excitation wavelength was set at 450 nm under which both FITC and Ru(phen)_3_^2+ ^present reasonable extinction coefficients higher than 10^4 ^M^-1 ^cm^-1 ^(see Figure S3 of Additional file 1) [23,24]. At pH = 2, the emission of FITC around 520 nm was quenched greatly. With the increased pH, the emission intensity of FITC increased gradually and then kept almost unchanged at pH ≥8, which was consistent with the behaviors of free FITC in aqueous solutions (see Figure S4 of Additional file 1). At the same time, the emission intensity of the reference dye located in the core part of the particles kept almost constant under the different pH. After being dispersed in BR buffers with pH of 2 to 8, the particles showed tunable emission color from red to yellowish-green which could be readily distinguished by naked eye under a 365-nm UV lamp (see insert of Figure 2). It is known that FITC may exist in dianionic, monoanionic, cationic, or neutral form dependent on pH of the solution (see Figure 2B). The monoanionic and neutral forms could be transformed into the non-luminous ester-type structure [25,26]. The pH-sensitive emission of the co-doped silica particles was primarily related to the equilibrium of FITC between the low quantum yield monoanionic form (*φ *= 0.36) and high quantum yield dianionic one (*φ *= 0.93). When pH of the solution was lowered, the emission intensity of FITC decreased greatly mainly attributed to formation of the non-luminous ester-type structure since there was no great difference in molar extinction coefficients of the momoanionic and dianionic forms (see Figure S4 of Additional file 1). Therefore, the particles showed a yellowish-green color at high pH and red color at low pH since the emission intensity of the red reference dye was almost insensitive to the changes in pH of the buffers.

**Figure 3:**
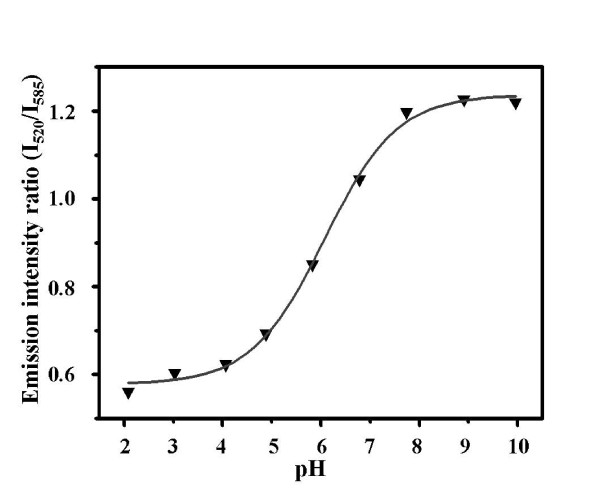
**Ratiometric calibration curve of the co-doped silica particles**. Based on the ratios of the emission intensity (*I*_520_/*I*_585_) of FITC and Ru(phen)_3_^2+ ^under different pH.

The variations in emission properties of the co-doped silica particles with pH could be further understood by the ratiometric calibration curve. Figure 3 shows the ratios in emission intensity (*I*_520/_/*I*_585_) of FITC (520 nm) and Ru(phen)_3_^2+ ^(585 nm) in the co-doped silica particles dispersed in BR buffers with different pH value. Emission intensity of both the two dyes was obtained from the same spectrum, which made the detection process become more convenient. The calibration curve followed the typical behavior of a system in equilibrium between the mono- and dianionic states of FITC. It is noted that the ratio increased rapidly in the range of pH from pH 5 to 8, attributed to the smart change in ratio of the mono- and dianionic forms of FITC around its pKa (6.4) [27].

**Figure 4:**
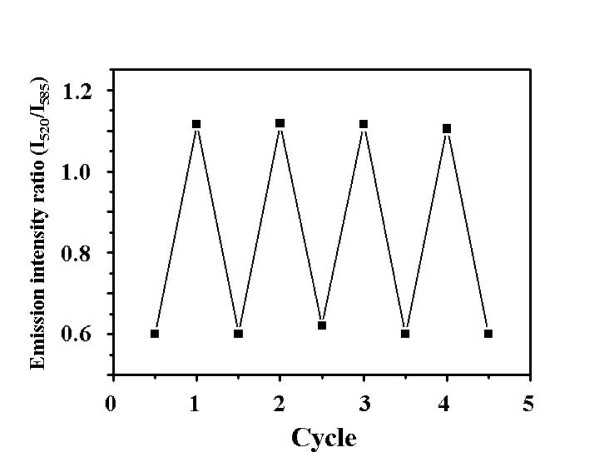
**Variations in the emission intensity ratios (*I*_520_/*I*_585_) of the co-doped silica particles**. Recoded at the start pH (pH = 4) and end pH (pH = 8) of different cycles. The excitation wavelength was at 450 nm.

Reversibility of the pH indicator was evaluated by monitoring the changes of the ratios in emission intensity of the two dyes (Figure 4). The co-doped silica particles were dispersed alternatively in BR buffers with pH 4 and 8. The ratio could be completely recovered when the particles were transferred between the BR buffers with pH 4 and 8. In addition, no leakage of the dyes from the particles was detectable even after 4 cycles. These results indicated that such co-doped silica particles are a kind of reversible and robust ratiometric pH indicator. It should be mentioned that the response of such ratiometric pH indicator was very fast (a couple of seconds), which may benefited from the efficient contact of the pH sensitive dye located on the particle surface with the analyte.

**Figure 5:**
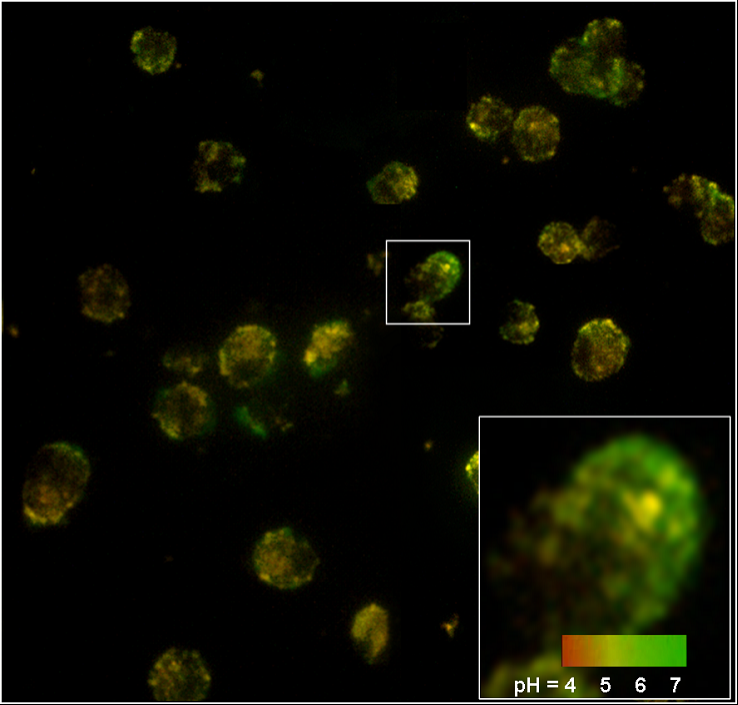
**Fluorescence image of the SMMC-7721 hepatoma cells after endocytosis of the co-doped silica particles**. The excitation wavelength was at 450 nm and the magnification was 400.

As mentioned above, the co-doped silica particles presented more sensitive response to pH around the pKa of FITC (6.4), meaning such pH indicator is suitable for detection of physiological pH. The nanoparticles were used to detect the intracellular pH micro-environment of SMMC-7721 hepatoma cells. TEM observations showed that the particles could be endocytosed and distributed in different compartments of the cells (see Figure S5 of Additional file 1). Figure 5 gives the image of the cells after endocytosis of the particles observed by a fluorescence microscopy. The particles showed distinguishable color even in one cell, corresponding to the different pH circumstance of the intracellular compartments. It was likely that the yellow color came from the particles internalized by lysosome, a kind of organelle with pH around 5, while the green color was contributed by the particles located in the cytoplasm and other organelles with neutral pH.

## Conclusion

In summary, visualized ratiometric pH indicator was fabricated by using a fluorescent dye (FITC) and a phosphorescent dye (Ru(phen)_3_^2+^). The two dyes were introduced into silica particles in a core/shell architecture to maximize the contact of the pH sensitive dye FITC with analyte while protecting the reference dye Ru(phen)_3_^2+ ^from the environment. Such ratiometric pH indicator could be excited simultaneously by using single wavelength due to the large overlapping in absorption features of the two dyes. The co-doped silica particles were sensitive to pH in the range of 2 to 8 distinguishable either by the emission spectra or in color observable by naked eye. The pH indicator showed good sensitivity around physiological pH, making it potential useful as a simple visualization pH indicator from detection of intracellular micro-environment.

## Abbreviations

FITC: fluorescein isothiocyanate; Ru(phen)_3_^2+^: tris(1, 10-phenanathroline) ruthenium ion; APS: 3-aminopropyltriethoxysilane; TEOS: tetraethoxysilane; DMEM: Dulbecco's Modified Eagle Medium; FBS: fetal bovine serum; PBS: phosphate-buffered saline; TEM: transmission electron microscopic.

## Authors' contributions

The work presented here was carried out in collaboration between all authors. JX carried out the laboratory experiments, interpreted the results, and drafted the paper. LS performed the cell experiments. JL, JlL, HM, and WY co-designed the experiments, discussed the experimental results, and revised the paper. All authors have contributed to, seen, read, and approved the manuscript.

## Competing interests

The authors declare that they have no competing interests.

## Supplementary Material

Additional file 1 **Supplementary data**. FITC and Ru(phen)_3_^2+ ^co-doped silica particles as visualized ratio-metric pH indicator. Figures S1 to S5. Supplementary data(1364224948562217).doc, 1110K. http://www.nanoscalereslett.com/imedia/2079142155617130/supp1.docSupplementary data files Click here for file
