# Supplementary material for: FITC and Ru(phen)32+ co-doped silica particles as visualized ratiometric pH indicator
Source: Nanoscale Res Lett. 2011 Oct 25;6(1):561. doi: 10.1186/1556-276X-6-561 (PMC3220666; doi:10.1186/1556-276X-6-561)
Supplement: Additional file 1 — Supplementary data. FITC and Ru(phen)32+ co-doped silica particles as visualized ratio-metric pH indicator. Figures S1 to S5. Supplementary data(1364224948562217).doc, 1110K. http://www.nanoscalereslett.com/imedia/2079142155617130/supp1.docSupplementary data files [file 1556-276X-6-561-S1.DOC]

**Supplementary data**

FITC and Ru(phen)_3_^2+^ Co-doped Silica Particles as Visualized Ratio-metric pH Indicator

Jianquan Xu, Lei Sun, Jun Li, Jinglun Liang, Huimao Zhang, and Wensheng Yang

**Figure S1.** Absorption spectra of the co-doped silica particles dispersed in DMF. DMF was chosen in order to closely match the refractive index of the solvent (1.43) to that of SiO2 particles (1.45), and thus to reduce the influence of scattering on the absorbance measurements. (see Ref. 22 in the text). The absorbance intensity of the peaks around 450 nm (Ru(phen)_3_^2+^) and 490 nm (FITC) could not be used to estimate the actual ratio of the two dyes because of the large overlapping in their absorption features.

**Figure S2.** Absorption spectra of the FITC doped silica particles (solid line) and the pure FITC (dotted line) dissolved in 0.5 M NaOH. To prepare the FITC doped silica particles, 52 nm pure silica particles without Ru(phen)_3_^2+^ was prepared and then FITC was grafted on the particle surface according to the procedures described in the experimental section. The particles were centrifuged and washed with water until the supernatant was clear. Then the particles were dissolved in 60 mL 0.5 M NaOH solution to liberate the dye molecules. 1 mg FITC was dissolved in 60 mL 0.5 M NaOH to prepare the pure FITC solution. The absorption spectra of the dissolved particle solution and the pure FITC solution were taken after the solutions were diluted for 10 times and the labeling efficiency was determined to be about 47% by comparing the absorbance intensity of the two samples at 490 nm.

**Figure S3**. Normalized absorption and emission spectra of Ru(phen)_3_^2+^ (solid lines: a, d) and FITC (dashed lines: b, c) in ethanol. The molar coefficient of FITC at the maximum absorbance (490 nm) is 8.7×10^4^ M^-1^ cm^-1^, which is sensitive to pH of the solution (see Ref. 23 in the text) and that of Ru(phen)_3_^2+^ at the maximum absorbance (~450 nm) is around 1.5×10^4^ M^-1^ cm^-1^, which is less sensitive to pH of the solution (see Ref. 24 in the text) .

**Figure S4.** Absorption (red) and emission (blue) spectra of FITC in BR buffers with pH of 2.1, 3.3, 4.1, 4.9, 5.8, 6.8, 7.8, 8.9, and 9.9. Based on the absorption spectra, the molar extinction coefficients of FITC at 450 nm increased slightly from 1.6×10^4^ M^-1^ cm^-1^ to 3.0×10^4^ M^-1^ cm^-1^ when pH of the solution increased from 2.1 to 9.9. However, the emission intensity of FITC at pH 9.9 was 100 times of that at pH 2.1, meaning that the change in emission intensity is mainly attributed to the equilibrium of FITC between the low quantum yield monoanionic form (φ = 0.36) and high quantum yield dianionic one (φ = 0.93) since there was no great difference in molar extinction coefficients of the momoanionic and dianionic forms.

**Figure S5** TEM images of SMMC-7721 hepatoma cells after endocytosis of the co-doped silica particles. To prepare the cell samples for TEM observations, first the cells were treated with the silica particles (100 μg/ml) for 3 h. Then the cells were washed with PBS and centrifuged at 2000 r/min for 10 min. After removal of the supernatants, the cell pellets were fixed in a 0.1 M PBS solution containing 2.5% gluteraldehyde and 4% paraformaldehyde for 3 h. After being washed with 0.1 M PBS, the cells were embedded in 2% agarose gel, and then post-fixed in 4% osmium tetroxide solution for 1 h. Then the cells were washed with distilled water, and stained with 0.5% aqueous solution of uranyl acetate for 1 h. After dehydration in ethanol, the cells were embedded in epoxy resin and kept at 60 °C for 48 h to promote polymerization of the resin. Ultra-thin sections obtained with a ultramicrotome were stained with 5% aqueous solution of uranyl acetate for 10 min and then 2% aqueous solution of lead citrate for 10 min before carrying out TEM measurements.
